# Videourodynamic findings of lower urinary tract dysfunctions in men with persistent storage lower urinary tract symptoms after medical treatment

**DOI:** 10.1371/journal.pone.0190704

**Published:** 2018-02-20

**Authors:** Yuan-Hong Jiang, Chung-Cheng Wang, Hann-Chorng Kuo

**Affiliations:** 1 Department of Urology, Buddhist Tzu Chi General Hospital and Tzu Chi University, Hualien, Taiwan; 2 Department of Urology, En Chu Kong Hospital, College of Medicine, National Taiwan University, New Taipei, Taiwan; 3 Department of Biomedical Engineering, Chung Yuan Christian University, Taoyuan, Taiwan; University Medical Center Utrecht, NETHERLANDS

## Abstract

**Objective:**

To analyze the underlying lower urinary tract dysfunctions by video-urodynamic studies in men who have persistent storage symptoms after initial drug therapy for lower urinary tract symptoms (LUTS) suggestive of benign prostatic hyperplasia (BPH).

**Methods:**

The medical records of 614 men **≥**40 years of age with LUTS and an International Prostate Symptom Score of **≥**8 were retrospectively analyzed. All patients had persistent storage symptoms after medical treatment for at least 6 months. A video-urodynamic study was done to investigate the underlying bladder or bladder outlet dysfunction. Predictors of bladder outlet obstruction (BOO) by baseline urine flow metrics and prostate parameters were investigated.

**Results:**

The final results revealed bladder neck dysfunction (BND) in 137/614 (22.3%), benign prostatic obstruction (BPO) in 246/614 (40.1%), detrusor overactivity (DO) in 193/614 (31.4%), and DO with detrusor underactivity (DO+DU) in 38/614 (6.2%) patients. Among the patients, 221/281 (78.6%) with a total prostatic volume (TPV) ≥40 ml had BOO, including 43/281 (15.3%) with BND and 178/281 (63.3%) with BPO. If we combined TPV ≥40 ml and Qmax <12 ml/s as predictors of BOO, BOO was found in 176/215 (81.8%) patients including 34/215 (15.8%) with BND and 142/215 (66.0%) with BPO. BOO was also found in 48.8% of men with a TPV <40ml, and in 36.3% of men with TPV< 40 ml and Qmax ≥ 12 ml/s. In 102 men with TPV <40 ml and Qmax ≥12 ml/s, 64 (62.7%) had DO.

**Conclusion:**

BOO, including BND and BPO, comprise 62.4% (383/614) of men with persistent storage symptoms after initial medical treatment for LUTS/BPH. In men who have persistent storage symptoms after medical treatment for LUTS/BPH, BOO should be carefully investigated and appropriate management being given to improve LUTS.

## Introduction

Lower urinary tract symptoms (LUTS) are highly prevalent in aged men [1]. AUA and EAU guidelines recommend to treat LUTS in men with an alpha-blocker alone or in combination with an alpha-reductase inhibitor when the total prostate volume (TPV) is greater than 30–40 mL [2,3]. About three-quarters of men with symptomatic bladder outlet obstruction (BOO) have LUTS improved with alpha-blocker monotherapy. The residual storage LUTS after alpha-blocker treatment is usually attributable to bladder dysfunction such as detrusor overactivity (DO) and an antimuscarinic is advised to add [2,4].

Recently, bladder dysfunction in men such as DO and detrusor underactivity (DU) have been shown to play important roles in LUTS [5]. The initial medical treatment with a combination of an alpha-blocker and an antimuscarinic or beta-3 adrenoceptor agonist has been recommended in treating men with LUTS suggestive of benign prostatic hyperplasia (LUTS/BPH) with predominantly storage symptoms [6,7]. It is likely that storage symptoms may exist primarily or secondarily to BPH or BOO; therefore, combined treatment is beneficial and can relieve LUTS after the initial medical treatment [4,8].

In clinical practice, it is common to encounter a group of men with mixed voiding and storage LUTS. After initial treatment for LUTS/BPH, the voiding symptoms improve, but storage symptoms persist. Recent studies have proven combined alpha-blocker and antimuscarinic drugs, or an antimuscarinic drug alone, often provide improvement for these patients [6,8]. Use of the IPSS voiding to storage subscore ratio has been proposed as a guide for initial treatment of men with mixed voiding and storage symptoms [9]. For patients in whom the initial treatment fails to improve the storage LUTS, it is possible that the initial diagnosis might be incorrect or the initial medication not powerful enough, leading to a suboptimal treatment outcome. In order to obtain the actual pathophysiology for the persistent storage LUTS, a urodynamic study is mandatory to identify the underlying pathophysiology and optimize a therapeutic strategy [5,10,11].

The role of urodynamic studies in men with LUTS and BPH is still controversial because the procedure is considered invasive and the results lack clinical significance [10]. Although several non-invasive tests have been proposed to replace urodynamic studies, measuring urodynamic pressure flow remains the gold standard test for the diagnosis of BOO [11]. The AUA and EAU guidelines recommend pressure flow studies as an optional test if patients with LUTS and BPH and are planning to undergo surgery [12,13]. Transurethral resection of the prostate (TURP) is not usually recommended unless BOO is proven by urodynamic pressure flow studies [14].

The aim of this retrospective study was to analyze the underlying lower urinary tract dysfunctions by video-urodynamic studies in men who have persistent storage symptoms after initial drug therapy for LUTS/BPH. We also investigated the possible relationship between TPV and the maximum flow rate (Qmax) and the diagnosis of bladder dysfunction and BOO such as bladder neck dysfunction (BND) and benign prostatic obstruction (BPO). The results can provide evidence for physicians to select appropriate men with persistent storage LUTS for surgical intervention and early symptom relief.

## Materials and methods

This study included men greater than 40 years of age with LUTS and an International Prostatic Symptom Score (IPSS) of equal or greater than 8. The data were retrospectively collected from the medical records from the years 2000 through 2014. Only patients with a moderate IPSS and a quality of life index greater than 3 were included in this study [15]. LUTS data included the IPSS voiding subscore (IPSS-V), storage subscore (IPSS-S), and voiding to storage subscore ratio (IPSS-V/S). All patients underwent transrectal sonography of the prostate to measure the TPV and determine the transition zone index (TZI) and measurement of urine flow metrics to determine the Qmax, voided volume, and post-void residual (PVR) volume. Based on the guidelines, patients received initial treatment with an alpha-blocker (in patients with TPV <40 ml) or a combined alpha-blocker and 5-alpha-reductase inhibitor (in patients with TPV ≥40 ml) for at least 6 months, but the storage symptoms (particularly urgency with or without urgency urinary incontinence) remained. Patients with an elevated prostatic specific antigen level of >4 ng/ml underwent a 12-core prostate biopsy to exclude the possibility of prostate cancer. The men also underwent a video-urodynamic study to investigate any underlying bladder or bladder outlet dysfunction.

Patients with frank urinary retention, PVR >200 ml, bladder stone, clinically overt BPO, or neurologic diseases such as cerebrovascular accident, Parkinson’s disease or spinal cord injury were not included in this study. The Ethics Committee of Buddhist Tzu Chi General Hospital approved this study (IRB: 104-15-B). Informed consent was waived due to the retrospective nature of the data collection. No patient had an active urinary tract infection before the examination.

The terminology and techniques of the urodynamic study was performed according to the recommendations of the International Continence Society (ICS) [16]. A video urodynamic study was used in this study for detailed investigation of the subtypes of BOO. The urodynamic parameters of the first sensation of filling, full sensation, bladder compliance, Qmax, PVR, voided volume, voiding detrusor pressure at Qmax (P_det.Qmax_), presence of DO, voiding efficacy (defined as voided volume/total bladder capacity), bladder contractility index (BCI, defined as P_det.Qmax_ + [5 x Qmax]), BOO index (defined as Pdet.Qmax—[2 × Qmax]) were measured and recorded in detail. During video-urodynamic study, the test was repeated at least twice to obtain reproducible pressure flow tracings.

**Fig 1:**
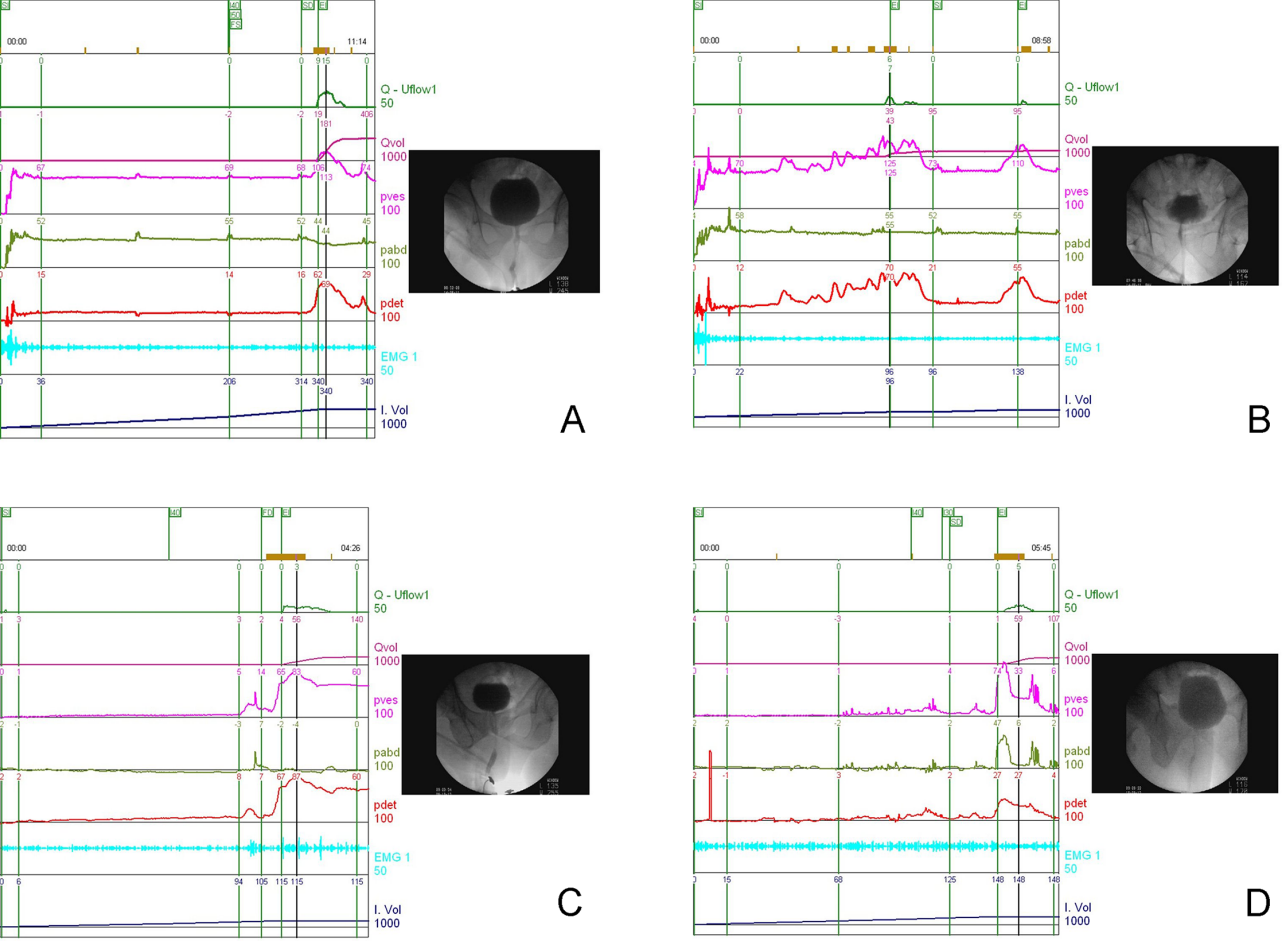
The videourodynamic tracings in men with (A) a stable bladder with normal detrusor pressure normal flow rate and open bladder outflow; (B) detrusor overactivity, high detrusor pressure and low flow rate, and tight bladder neck and open prostatic urethra during voiding, suggestive of bladder neck dysfunction; (C) phasic detrusor overactivity, high detrusor pressure and low flow rate, with a narrow prostatic urethra during voiding, suggestive of prostatic obstruction; (D) phasic and terminal detrusor overactivity, and open bladder outflow during voiding, suggestive of detrusor overactivity and detrusor underactivity without bladder outlet obstruction.

The lower urinary tract conditions such as BPO, BND, poor relaxation of the external sphincter (PRES), and DU during voiding phase, and increased bladder sensation and DO during filling phase were diagnosed according to the findings of characteristic bladder outlet and bladder dysfunction during the study [5,16]. The definition of BOO was based on the provisional ICS definition of obstruction characterized by a high detrusor pressure and a reduced Qmax.^16^ In this study, BOO was defined as a BOOI greater or equal to 40, or a pressure-flow study showing a P_det.Qmax_ greater or equal to 50 cmH_2_O. In patients with equivocal pressure flow results, the features of the bladder neck, prostatic urethra, and external sphincter on voiding cystourethrography were used for the diagnosis of BOO [5]. BPO was diagnosed if the prostatic urethra was narrow during the voiding phase. BND was diagnosed if the bladder neck did not open to a funnel shape during voiding, regardless of whether the voiding P_det.Qmax_ was high or normal. DO was defined as urodynamic evidence of spontaneous detrusor contraction occurring during bladder filling (phasic DO) or occurring before uninhibited detrusor contraction at bladder capacity (terminal DO). When patient was found to have DO during filling and associated with incomplete bladder emptying with a VE of less than 67% or PVR of more than 100mL, a diagnosis of DO+DU was used in this study. Patients with DO or DO+DU all showed open bladder neck and prostatic urethra during voiding. Patients with proven BND or BPO might have concomitant phasic or terminal DO. PRES was diagnosed if the urethral sphincter electromyography (EMG) showed no relaxation of a narrow membranous urethra during voiding [17]. However, when the membranous urethral sphincter EMG showed good relaxation during voiding, a low flow rate was considered to be due to DU. Fig 1 demonstrates the video-urodynamic tracings of patients with normal bladder outflow, BND, BPO and DO+DU.

Continuous variables are represented as means ± standard deviations (SDs), and categorical data are represented by number and percentage. Statistical comparisons between the groups were tested using the chi-square test for categorical variables, and the Wilcoxon rank-sum test for continuous variables. Statistical assessments were considered significant when *p* was < 0.05. Statistical analyses were performed using SPSS 15.0 statistical software (SPSS Inc., Chicago, IL).

## Results

The analysis included 614 men. All of them had symptoms of urgency with or without urgency urinary incontinence after the initial medical treatment for LUTS/BPH. The final video-urodynamic study revealed BND in 137 (22.3%), BPO in 246 (40.1%), DO in 193 (31.4%), and DO+DU in 38 (6.2%) patients. Nearly two-thirds (62.4%) of the men still had BOO on video-urodynamic studies after initial medical treatment for LUTS/BPH.

Table 1 presents the patients’ age, IPSS, prostatic measures, and urodynamic parameters. Patients with DO+DU were significantly older than were those with BND, BPO, and DO. LUTS symptom scores including IPSS-total, IPSS-subscore, and V/S ratio showed no significant differences between the groups. Patients with BPO had significantly larger TPV and TZI than those in the other groups. Urodynamic studies showed patients with BPO had the highest Pdet, followed by those with BND, DO, and DO+DU. Qmax was significantly greater in patients with DO and did not differ significantly among patients with BND, BPO, and DO+DU. PVR was significantly smaller in DO group compared with that in BND and BPO groups and was larger in the patients with DO+DU. Urodynamic DO was observed in most of the patients. BOOI was the greatest in the BPO group, and BND ranked second and was the lowest in the DO and DO+DU groups.

**Table 1:** Variables among men with lower urinary tract dysfunction with storage symptoms after medical treatment for LUTS or BPH.

	BND (n = 137)	BPO (n = 246)	DO (n = 193)	DO + DU (n = 38)	ANOVA
**Age**	71.6 ± 10.6	74.3 ± 8.55	73.3 ± 9.70	79.0 ± 7.91	0.0001
**IPSS total**	12.4 ± 7.69	13.7 ± 8.23	15.1 ± 9.27	18.0 ± 6.40	0.213
**IPSS voiding**	6.42 ± 5.85	6.94 ± 5.75	7.63 ± 6.56	10.7 ± 4.39	0.306
**IPSS storage**	6.27 ± 3.57	6.61 ± 3.83	7.84 ± 3.98	7.29 ± 2.50	0.131
**IPSS-V/S**	1.22 ± 1.42	1.29 ± 1.27	1.16 ± 1.49	1.49 ± 0.53	0.898
**TPV (mL)**	36.6 ± 16.5	62.8 ± 35.7	34.4 ± 17.2	36.0 ± 18.1	0.0001
**TZI (%)**	35.8 ± 11.2	48.1 ± 12.4	34.5 ± 17.2	36.0 ± 18.1	0.0001
**FSF (mL)**	112 ± 54.3	106 ± 54.6	110 ± 56.9	124 ± 65.0	0.282
**FS (mL)**	166 ± 78.8	152 ± 78.6	160 ± 81.7	186 ± 88.4	0.054
**Compliance**	50.5 ± 54.6	46.6 ± 52.8	54.0 ± 62.3	53.5 ± 55.9	0.571
**Pdet(cmH**_**2**_**O)**	48.2 ± 24.8	71.3 ± 25.5	31.8 ± 12.8	20.3 ± 11.3	0.0001
**Qmax (mL/s)**	8.59 ± 4.40	7.55 ± 4.39	11.9 ± 5.31	5.95 ± 3.70	0.0001
**Volume (mL)**	201 ± 110	169 ± 97.9	222 ± 107	117 ± 77.6	0.0001
**PVR (mL)**	55.9 ± 82.3	63.5 ± 85.5	19.4 ± 41.9	161 ± 106	0.0001
**UDS DO**	115 (83.8%)	224(91.1%)	193 (100%)	38 (100%)	0.750
**BOOI**	31.0 ± 27.6	56.2 ± 27.4	7.98 ± 16.4	8.40 ± 12.8	0.0001
**VE (%)**	79.2 ± 28.9	75.4 ± 27.0	92.9 ± 14.0	45.6 ± 24.1	0.0001
**BCI**	91.1 ± 20.4	109 ± 32.8	91.2 ± 29.9	50.0 ± 22.6	0.0001

BND, bladder neck dysfunction; BPO, benign prostatic obstruction; UDS, urodynamics; DO, detrusor overactivity; DHIC, detrusor hyperactivity and inadequate contractility; IPSS, International Prostatic Symptom Score; IPSS-V/S: IPSS voiding to storage ratio; TPV, total prostatic volume; TZI, transitional zone index; FSF, first sensation of filling; FS, full sensation; Pdet, detrusor pressure; Qmax, maximum flow rate; PVR, post-void residual volume; BOOI, bladder outlet obstruction index; VE, voiding efficiency; BCI, bladder contractility index.

When we used TPV as the predictor for the differential diagnosis of lower urinary tract dysfunction in the study patients, 221 (78.6%) patients with TPV ≥ 40 ml had BOO including 43 (15.3%) with BND and 178 (63.3%) with BPO. Another 162 (48.8%) patients with TPV <40 ml had BOO including 94 (28.3%) with BND and 68 (20.5%) with BPO. When we used Qmax as the predictor, 301 (67.6%) patients with a Qmax <12 ml/s had BOO including 105 (23.6%) with BND and 196 (44.0%) with BPO. BOO can still be found in 48.5% of men with Qmax ≥ 12 ml/s (Table 2). If we combined TPV ≥40 ml and Qmax <12 ml/s as predictors, BOO was found in 176 (81.8%) patients including 34 (15.8%) with BND and 142 (66%) with BPO. In 102 patients with TPV <40 ml and Qmax ≥12 ml/s 64 (62.7%) had DO, while 37 (36.3%) had BOO (Table 3).

**Table 2:** Relationship of video-urodynamic diagnosis based on total prostatic volume and maximum flow rate.

	Total no.	BND (n = 137) No. (%)	BPO (n = 246) No. (%)	DO (n = 193) No. (%)	DO+DU(n = 38) No. (%)
**TPV< 40 mL**	332	94 (28.3) (68.6)	68 (20.5) (27.6)	145 (43.7) (75.1)	25 (7.5) (65.8)
**TPV ≥ 40 mL**	281	43 (15.3) (31.4)	178 (63.3) (63.4)	48 (17.1) (24.9)	13 (4.6) (34.2)
**Qmax <12mL/s**	445	105 (23.6) (76.6)	196 (44.0) (79.7)	108 (24.3) (56.0)	36 (8.1) (94.7)
**Qmax ≥12mL/s**	169	32 (18.9) (23.4)	50 (29.6) (20.3)	85 (50.3) (44.0)	2 (1.2) (5.3)

BND, bladder neck dysfunction; BPO, benign prostatic obstruction; DO, detrusor overactivity; DHIC, detrusor hyperactivity and inadequate contractility; TPV, total prostatic volume; Qmax, maximum flow rate.

**Table 3:** Relationship of video-urodynamic diagnosis and combination of total prostatic volume and maximum flow rate.

	Total no.	BND (n = 137) No. (%)	BPO (n = 246) No. (%)	DO (n = 193) No. (%)	DO+DU(n = 38) No. (%)
**TPV <40 mL & Qmax <12 mL/s**	230	71 (30.9) (51.8)	54 (23.5) (22.0)	81 (35.2) (42.0)	24 (10.4) (63.2)
**TPV <40 mL & Qmax ≥12 mL/s**	102	23 (22.5) (16.8)	14 (13.7) (5.7)	64 (62.7) (33.2)	1 (1) (2.6)
**TPV ≥40 mL & Qmax <12 mL/s**	215	34 (15.8) (24.8)	142 (66) (57.7)	27 (12.6) (14.0)	12 (5.6) (31.6)
**TPV≥ 40 mL & Qmax ≥12 mL/s**	67	9 (13.4) (6.6)	36 (53.7) (14.6)	21 (31.3) (10.9)	1 (1.5) (2.6)

BND, bladder neck dysfunction; BPO, benign prostatic obstruction; DO, detrusor overactivity; DHIC, detrusor hyperactivity and inadequate contractility; TPV, total prostatic volume; Qmax, maximum flow rate.

The differential diagnosis of BND and BOO was not easy. About half of the patients with BND had TPV <40 ml and Qmax <12 ml/s. However, these parameters were not significantly different from patients with DO or DO+DU. One-quarter of the patients with BND also had Qmax ≥12 ml/s. A diagnosis based on the video-urodynamic study seemed to be accurate for detecting BND in patients with OAB symptoms after medical treatment for LUTS or BPH.

## Discussion

This study revealed in men with persistent storage LUTS after initial medical treatment for LUTS/BPH, the diagnosis of significant obstruction can be identified on the basis of increased TPV and decreased Qmax. A TPV > 40 ml predicts BOO in 78.6% of men and a TPV > 40 ml with Qmax < 12 ml/s predicts BOO in 81.8% of men with persistent storage LUTS after initial treatment. BOO was also found in 48.8% of men with a TPV < 40 ml, and in 36.3% of men with TPV < 40 ml and Qmax ≥ 12 ml/s.

The results emphasize that LUTS does not correlate with bladder or bladder outlet dysfunction, and prostate size does not relate to symptoms or BOO adequately, especially in men with persistent storage LUTS after initial treatment for LUTS/BPH. Although DO and DO+DU are important lower urinary tract dysfunctions in this cohort, BOO should not be overlooked because surgical intervention may alleviate storage symptoms. However, an accurate diagnosis of BOO should be made if we intend to undertake surgical intervention in men with persistent storage symptoms after medical treatment for LUTS/BPH.

LUTS are highly prevalent in men, and the incidence increases with age [1]. In the past, LUTS are usually considered as synonyms of BPH; however, only 25% to 50% of men with BPH have LUTS, and urodynamically proven BOO is only seen in 50% of men with LUTS [18]. Many clinical studies have demonstrated that LUTS have poor diagnostic specificity for BOO, and in some of the patients with LUTS/BPH symptoms do not improve after TURP [19]. Although an enlarged prostate might not indicate the presence of BOO, the mean TPV of patients with BOO is significantly greater than that of patients without BOO [20]. Patients with persistent LUTS after TURP are found to have a small TPV at the time of surgery, suggesting that a non-BPH etiology could account for their LUTS [21]. Therefore, the clinical diagnosis of BPH should be carefully considered, especially when an invasive procedure is going to be performed.

The focus on LUTS has recently shifted from the prostate to the bladder [22]. Several investigators suggest that not all men with LUTS have associated prostate pathology or BOO, and thus bladder dysfunction plays a role in the pathogenesis of LUTS. Nonetheless, it is difficult to distinguish the causes of LUTS in men based on the clinical symptoms, and a subset of patients receiving medical treatment for prostate conditions may have residual storage symptoms [4,14]. The causes for the persistent storage symptoms after initial medical treatment for LUTS/BPH could be too high expectation of the patients, too low dose of medication for high grade BOO, or inaccurate initial diagnosis of LUTS. In this study, we found that 62.4% of patients with persistent storage symptoms after initial treatment still had BOO, whereas the remaining 37.6% had DO or DHIC. These results suggest a large portion of patients initially treated as LUTS/BPH could be undertreated or wrong diagnosed. Adding 5ARI on alpha-blocker in patient with BPO and a TPV<40 ml may improve BOO condition. TUIP or TURP might be beneficial for patients with BND or BPO proven by video-urodynamic study, but will be ineffective in those with DO or DHIC.

The diagnostic rationale of urodynamic studies in association with the currently changing management paradigm of lower urinary tract dysfunction has been debated for a long time. Surgical intervention to relieve BOO and LUTS, based on a urodynamic diagnosis, results in an improvement of flow rate after TURP. ^10^ In patients with storage symptoms and empiric treatment failure, urodynamic pressure flow studies can provide definitive information that can identify associated pathologies and alter the treatment course [23].

This study revealed that TPV remains a significant predictor for men with persistent storage symptoms after medical therapy for LUTS/BPH. A TPV ≥40 ml predicted the presence of BOO in 78.6% of patients while combined TPV ≥40 ml and Qmax <12 ml/s predicted BOO in 81.8% of patients. In the clinics where video-urodynamic studies or pressure flow studies are not available, these predictive factors may be used for differential diagnosis of the presence of BOO. However, for the men with persistent storage LUTS after initial treatment, BOO can still be found in 48.8% of men with TPV <40 ml, and in 48.5% of men with Qmax ≥ 12 ml/s. Even in men with TPV < 40 ml and Qmax ≥ 12 ml/s, BOO can still be found in 36.3%. In patients who have TPV <40 ml and Qmax ≥12 ml/s, 62.7% of them had DO, and surgical intervention should be avoided in them to prevent undesired therapeutic outcomes. Patients who are older, with a small TPV and large PVR, a diagnosis of DO+DU should be carefully considered, and surgical intervention should be cautiously recommended.

Recent investigations have revealed that bladder dysfunction and bladder outlet dysfunction contribute equally to LUTS in men. In patients with a TPV of less than 40 ml, a diagnosis of bladder dysfunction such as increased bladder sensation, DO, or DU should be considered. In men with persistent storage symptoms after the initial medical treatment for LUTS/BPH, an antimuscarinic drug or beta-3 adrenoceptor agonist is recommended for treatment of DO or DO+DU [24,25]. Video-urodynamic studies should be performed for ascertaining the specific differential diagnosis of lower urinary tract dysfunction when patients still have storage symptoms. Only the men that do not respond to the combination medical treatment and that are proven to have BND or BPO should be referred for surgical intervention. A comprehensive video-urodynamic study will enable the correct selection of therapy aimed at the underlying pathophysiology.

This study has several limitations. First, this is a retrospective study and selection bias may exist. Second, these patients were not treatment-naïve and the parameters of urodynamic study may change after medical treatment. DO may disappear in patients with BPO after alpha-blocker treatment. However, one advantage of the present study is that the results can be easily applied to real world practice. Although possible differences in prostatic growth might exist between Asians and westerns, the results shown in this study may be globally representative. Urologist should avoid surgical intervention in patients with small prostate and high Qmax because DO is the most common diagnosis in these patients.

## Conclusion

Bladder outlet dysfunction such as BND and BPO are highly prevalent in men with persistent storage symptoms after medical treatment for LUTS/BPH. However, DO and DO+DU should not be overlooked, especially when an invasive surgical procedure is under consideration. The combination of TPV ≥40 ml and Qmax <12 ml/s is a strong predictive factor for BPO while a combination of TPV <40 ml and Qmax ≥12 ml/s suggests a high possibility of DO or DO+DU. In men who have persistent storage LUTS after medical treatment for LUTS/BPH, BOO should be carefully investigated and appropriate management being given to improve LUTS.
